# Genome-wide analysis of a avirulent and reveal the strain induces pro-tective immunity against challenge with virulent Streptococcus suis Serotype 2

**DOI:** 10.1186/s12866-017-0971-0

**Published:** 2017-03-14

**Authors:** Jing Wang, Youjun Feng, Changjun Wang, Feng Zheng, Bachar Hassan, Liming Zhi, Wenjuan Li, Yi Yao, Elaine He, Shibo Jiang, Jiaqi Tang

**Affiliations:** 1 Translational Medicine Center, PLA Hospital No. 454, Nanjing, 210002 China; 2 Department of Medical Microbiology and Parasitology, Zhejiang University School of Medicine, Hangzhou, Zhejiang 310058 China; 3 Department of Epidemiology, Medicinal Research Institute, Nanjing Military Command, Nanjing, 210002 China; 4 University of North Carolina, Chapel Hill, NC USA; 5 The Warren Alpert Medical School of Brown University, Providence, RI02912 USA; 6 PLA Research Institute of Clinical Laboratory Medicine, Nanjing General Hospital, Nanjing Military Command, Nanjing, 210002 China; 7 Lindsley F. Kimball Research Institute, New York Blood Center, New York, NY 10065 USA

## Abstract

**Background:**

It was previously reported in China that two recent large-scale outbreaks of *Streptococcus suis* serotype 2 (*S. suis* 2) infections in human were caused by two highly virulent *S. suis* 2 strains, from which a novel genomic island (GEI), associated with disease onset and progression and designated 89 K, was identified. Here, an avirulent strain, 05HAS68, was isolated from a clinically healthy pig.

**Results:**

By comparing the genomes of this avirulent strain with virulent strains, it was found that massive genomic rearrangements occurred, resulting in alterations in gene expression that caused enormous single gene gain and loss. Important virulent genes were lost, such as extracellular protein factor (*ef*) and suilysin (*sly*) and larger mutants, such as muramidase-released protein (*mrp*). Piglets vaccinated with the avirulent strain, 05HAS68, had increased TNF-α and IFN-γ levels in the peripheral blood and were fully protected from challenge infection with the most virulent *S. suis* 2 strain, 05ZYH33. Transfusion of T cells and plasma from vaccinated pigs resulted in protection of recipient animals against the 05ZYH33 challenge.

**Conclusion:**

These results suggest that analysis genome of the avirulent strains are instrumental in the development of vaccines and for the functional characterization of important of genetic determinants.

**Electronic supplementary material:**

The online version of this article (doi:10.1186/s12866-017-0971-0) contains supplementary material, which is available to authorized users.

## Background

The genus *Streptococcus* is one of the most diverse gram-positive pathogen for both humans and livestock. A few multivalent streptococcal conjugate vaccines have been introduced and have significantly reduced the invasiveness of these pathogens. Recently however, there are some strains that have become resistant to these new types of vaccine [1]. *S. suis* is one of the most serious pathogens, causing meningitis, septicemia, arthritis and other diseases in pigs [2] and is responsible for substantial economic losses in the swine industry. *S. suis* has also been associated with meningitis, endocarditis and other diseases in humans [3]. At present, 32 serotypes, based on capsular antigens, have been identified. Serotype 2 is considered the most virulent and is frequently isolated from both swine and humans [4]. The mechanisms involved in the pathogensis and virulence of serotype 2 (*S.suis* 2) are poorly understood. *S. suis* 2, followed closely by *S. pneumonia*, has been found to have the greatest number of gene gain and loss under positive selection pressure [5]. Prevention and control of diseases caused by this pathogen is hampered by the lack of an effective vaccine that generates a protective response.

Several candidate vaccines have been investigated. First, vaccines against bacteria that cause disease mainly by production of virulence protein factors were considered such as suilysin (SLY), muramidase-released protein (MRP) and extracellular protein factor (EF). These proteins have been shown to protect pigs from homologues and heterologous serotype 2 strains; however, a substantial number of virulent strains in some geographic regions do not express these proteins [6, 7]. Second, capsule polysaccharide is another type of virulence factor found in *S. suis*. However, a vaccine based on capsular material was unsatisfactory because polysaccharide and lipoligosaccharide antigens are similarly variable and are unlikely to provide long-lived immunity, since they are T-cell-independent antigens that are incapable of stimulating immunological memory [8]. Third, some immunity protection has been reported using killed whole cells; however the heat-killed bacteria are rapidly eliminated by chemokine-activated phagocytes and are unable to activate protective long-lasting or immunological memory responses [9]. *S.suis* vaccines, either polysaccharide, conjugate or killed bacteria, are currently being tested in trials in order to overcome the rise in worldwide antibiotic resistance, However, given the reasons stated above, none of these vaccines are expected to provide full immunological coverage in a near future. It is generally accepted that live-attenuated or avirulent vaccines are suitable for inducing cell-mediated immunity without affecting humoral immunity. Attenuated and/or avirulent live *S.suis* strains have been tested, though the results have been equivocal [10, 11]. This concern has prompted exploration into alternative possibilities with better therapeutic potentials against diseases caused by *S.suis*.

At present, the few known factors related to pathogenesis have not been found to induce global protection and are of limited use. Comparing and analyzing genome sequences between virulent and avirulent strains of *S.suis* 2 may predict ORFs, specify unique genes and reveal the presence of some previously uncharacterized genes predicted to involve in virulence and gene regulation. This discovery will provide strategies for vaccine development and new approaches in the prevention and treatment of diseases caused by *S.suis* 2.

In this study, an avirulent *S.suis* 2 strain, designated 05HAS68, was isolated from a healthy pig in the Jiangsu area of China. Genome analysis of strain should significantly increase our understanding of different virulence related genes and genomic islands of highly virulent strains, 05ZYH33 and 98HAH33, which come from the same affected areas. The genomic analysis will increase our understanding the pathogenesis and evolution of these strains. Using this information, we evaluated the ability of the avirulent strain, 05HAS68, to provide protective immunity against a challenge with *S.suis* 2 virulent strain, 05ZYH33, taken from a patient with streptococcal toxic shock syndrome (STSS).

## Methods

### Bacterial strains and growth

A single-colony isolate of the *S. suis* 2 strain was assigned the unique strain designation, 05HAS68. This isolate originated from clinically-healthy pigs in the affected areas of Jiangsu in China. This strain and the reference strain, 05ZYH33 were grown in Todd-Hewitt broth (THB) (Difco Laboratories, Detroit,MI) medium or and plated on THB agar plates containing 5% (vol/vol) sheep blood at 37 °C.

### Identification of *Streptococcus suis* serotype

Serotyping was performed by slide agglutination using specific antisera (Statens Serum Institute, Copenhagen, Denmark) and was positive for serotype 2. Briefly, 2 μl of the broth was mixed with 8 μl of 0.1 M PBS (pH 7.4) buffer containing 1: 500 diluted serotype-specific antibody or 3% BSA, incubated at room temperature for 30 min, and visualized at 400 × optical microscope.

### Experimental infections of piglets

Three-week-old male specific-pathogen-free (SPF) - piglets from the experimental animal center of Medicinal Research Institute of Nanjing Military Command, were divided into groups, six piglets per group, and were used to evaluate protective effect tests and or specific T cells transfer protective effect tests (6 piglets/group in random). The avirulent strain 05HAS68 was used as the experimental group and virulent strain 05ZYH33 and 98HAH33 was as positive control. All of the SPF piglets were inoculated intravenously at a dose of 1.8 × 10^9^ CFU per piglet through the ear with different bacterial suspensions. For the virulence studies, three groups of piglets were directly injected with 05HAS68, 05ZYH33, or 98HAH12, respectively. To evaluate the protective effect of 05HAS68, two groups of piglets were simultaneously inoculated with 05HAS68, and were given the same dose of bacterial suspensions for booster on day 7. Subsequently, these two groups were challenged with 05ZYH33 or 98HAH12 on 14. All animal experiments were performed in a biosafety level 3 facilities and approved by the ethical committee of PLA Hospital No. 454.

### Genome sequencing and comparative genomics

The 05HAS68 *S. suis* genome was sequenced and assembled using the routine random shotgun method [12]. Prediction of putative coding sequences (CDSs) and gene annotation were done using the NCBI’s Microbial Genome Annotation Tools and Genome Annotation Pipeline (http://www.ncbi.nlm.nih.gov/genome/browse/reference/). The predictions and the slices were then searched against the NCBI Entrez Protein Cluster database (https://www.ncbi.nlm.nih.gov/proteinclusters/browse/stats/) as well as proteins from all complete microbial genomes. The genome sequence feature information was visualized using the CGView genome visualization program ([13] http://stothard.afns.ualberta.ca/). Annotation was supplied from both sets and supplemented with information from the Conserved Domain Database (https://www.ncbi.nlm.nih.gov/cdd) and from Clusters of Orthologous Groups [14]. The protein sets of strains 98HAH33, P1/5 and 05HAS68 were compared using FASTA3. The sequence alignment was produced by Mauve Genome software [14] and the phylogenetic tree was built using CLC Genomics Workbench 3.2 software (https://www.qiagenbioinformatics.com/genomics/)*.* Comparison of *S. suis* genome structures was done as follows: pair wise comparisons of the P1/7, 05ZYH33 and 05HAS68 displayed chromosomes was done using the Artemis Comparison Tool (ACT) [15, 16] and a dot matrix was used to show the relatedness of genome sequences generated with MUMmer [17]. The 05HAS68, 98HAH33, 05ZYH33 and P1/7 CRISPR defense systems were analyzed using CRT1.1 software [18]. Identification of the bacterial CRISPR-associated proteins (Cas) adjacent to the CRISPR loci and comparative analysis of proteins from putative prophages, viruses (bacteriophage and viruses of archaea), and plasmid-like sequences was done using the Non-Redundant GenBank databases (https://www.ncbi.nlm.nih.gov/genbank/).

### Light and transmission electron microscopy (TEM)

To examine the strains morphological differences between 05HAS68 and 05ZYH33, the methods were performed according to our described previously [4]. The samples from agar-grown bacteria (05HAS68 and 05ZYH33 strain) were prepared by conventional methods. Briefly, bacterial cell were fixed in 5% glutaraldehyde for 2 h, post fixed with 2% osmium tetroxide for 2 h, dehydrated in a graded series id acetone washes, and embedded in Epon-812 epoxy resin. Thin sections were post stained with uranyl acetate and lead citrate and then examined with a JEM-1010 TEM (Jeol Ltd., Tokyo, Japan) at an accelerating voltage of 100KV.

### Proteome analysis

The *S. suis* strains, 05HAS68 and 05ZYH33, were grown at 37 °C in 400 mL THB, until they reached the mid-exponential phase (OD600 0.8). The bacterial cells were harvested by centrifugation at 5000 × *g* for 10 min and washed with phosphate-buffered saline. Samples were then homogenized on ice in 1 ml of lysis buffer (7 M urea, 2 M thiourea, 4% CHAPS, 30 mM Tris-Cl, pH 8.5, protease inhibitor mixture) using a glass homogenizer. After sonication on ice for 10 s using an ultrasonic processor, the samples were centrifuged for 30 min at 20,000 × g to remove particulate material. Aliquots (280 μl) of the samples were loaded onto 13-cm Immobiline DryStrip gels (pH 3 to 10; GE Healthcare Biosciences Co.). The first-dimensional electrophoresis conditions and second-dimensional sodium dodecyl sulfate- polyacrylamide gel electrophoresis separation were done as described previously. The separated proteins were transferred to PVDF membranes by electrotransfer. After transfer, membranes were blocked with 1% w/v BSA in PBS (pH 7.4) for 1.5 h at room temperature. The membranes were incubated with sera taken from swine during the convalescent-phase of 05ZYH33 infection or from swine that had been vaccinated with 05HAS68. The sera (1:1000 dilution) was incubated for 1 h at room temperature and subsequently washed three times with 0.05% w/v Tween-20 in PBS for 10 min. Immunodetection was performed with Staphylococcal Protein A-HRP (SPA-HRP, Boster; 1:1000 dilution) at room temperature. Before and after the addition of the secondary antibody, the membranes were washed three times with 0.05% w/v Tween-20 in PBS for 15 min. The membranes were then washed with 50 mM Tris-HCl buffer (pH 7.4) and developed with tetra hydrochloride (DAB, AMRESCO) substrate until optimum color development was observed.

To compare the abundance of protein spots on the 2-dimensional electrophoresis gels, the stained gels were scanned and analyzed with an Image-Master instrument. Each protein spot was analyzed using Malanie viewer 7 software (http://world-2dpage.expasy.org/melanie/).

### Lymphocyte preparation, culture and T cell adoptive transfer

The lymphocytes were isolated from the heparinized blood from SPF pigs vaccinated with the avirulent strain, 05HAS68. Briefly, lymphocytes (>85% purity) were prepared by sequential centrifugation on Ficoll and Percoll gradients. After isolation, cells (1 × 10^6^ cells/ml) were incubated with heat-inactive 05HAS68 cells (1 × 10^6^ cells/ml) for 3 days at 37 °C in a 5% CO2 atmosphere. The specific T lymphoblasts were prepared by sequential centrifugation on Ficoll gradients. After washing, the cells were resuspended in RPMI1640 and 1.8 × 10^9^ cells or mixed with 5 ml plasma from the piglets vaccinated with avirulent 05HAS68 and were then transferred to naïve piglets (*n* = 6) via injection into the ear vein.

### TNF-α and IFN-γ quantification by ELISA

Plasma or sera from the pigs was collected following centrifugation and stored at −80 °C until ELISA analysis. TNF-α and IFN-γ was quantified using a swine immunoassay kit (JINMEI BITECH, Beijing, China), as specified by the manufacturer. Sample dilutions giving optical density readings in the linear portion of the appropriate standard curve were used to quantify the level of each cytokine. Standard and sample dilutions were added to duplicate wells on each ELISA plate, and all analyses were performed at least three times for each individual stimulation assay. The A450 of the plates was determined using a microplate reader (Thermo Scientific Multiskan GO, USA). For all comparisons, was considered *P <* 0.05 to be significant. Statistical analyses were performed using SPSS 11.0 for Windows

### Statistical analysis

Quantitative results were expressed as the means ± SEM. The data were analyzed using IBM SPSS Statistics 19. The unpaired two-tailed Student’s t-test or Mann-Whitney test was used to compare means between different groups. One-way ANOVA with Bonferroni’s post-test was considered appropriate for multiple comparisons. *P*-values less than 0.05 were considered significant.

## Results

### Basic biological features

**Fig. 1:**
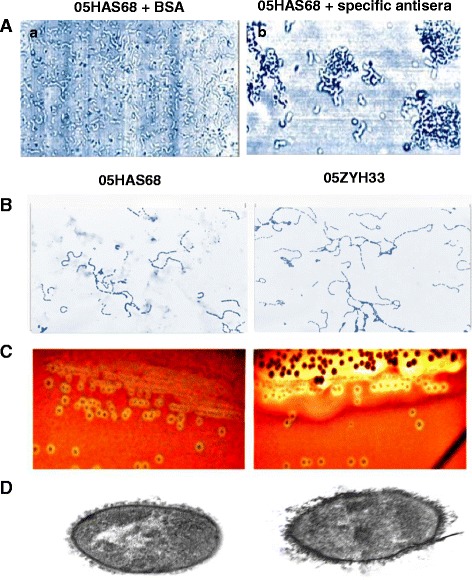
Identified as *S. suis* serotype 2 using an agglutination test and the biological features of 05HAS68. **a** Strain 05HAS68 serotype identification *a*. Strain 05HAS68 incubated with 3% BSA 0.1 M PBS as control; *b*. Strain 05HAS68 incubated with coagglutination reagent of specificanti-capsular type 2 antibody. Comparison of basic biological characteristics between strain 05HAS68 and 05ZYH33 (**b**-**d**): **b**), Light microscope morphology of *S. suis* strains using Gram-staining. **c** Hemolytic phenotype of the strains on blood agar plates. **d** Transmission electron micrographs of bacteria cultured in THS

*S. suis* strain 05HAS68, isolated from a healthy pig was identified by a quelling reaction and agglutination test. The bacterial body of the 05HAS68 strain showed agglutination and swelled after incubation with *S.suis* type 2 capsular antiserum (Fig. 1a). This result indicated that the 05HAS68 strain belongs to *S.suis* serotype 2. A slight difference in growth was observed between the virulent 05ZYH33 and avirulent 05HAS68 strains. The 05HAS68 strain grew at a slower rate than the 05ZYH33 strain in THB and the mean chain length of 05HAS68 was longer than that of 05ZYH33 under the same growth conditions (Fig. 1b). Hemolytic analysis on sheep blood agar plates indicated that 05HAS68 tends to induce diminutive hemolytic zones, compared with 05ZYH33 (Fig. 1c). Similar capsular thickness was observed between the 05HAS68 and 05ZYH33 strains (Fig. 1d).

### General genomic characterization

**Fig. 2:**
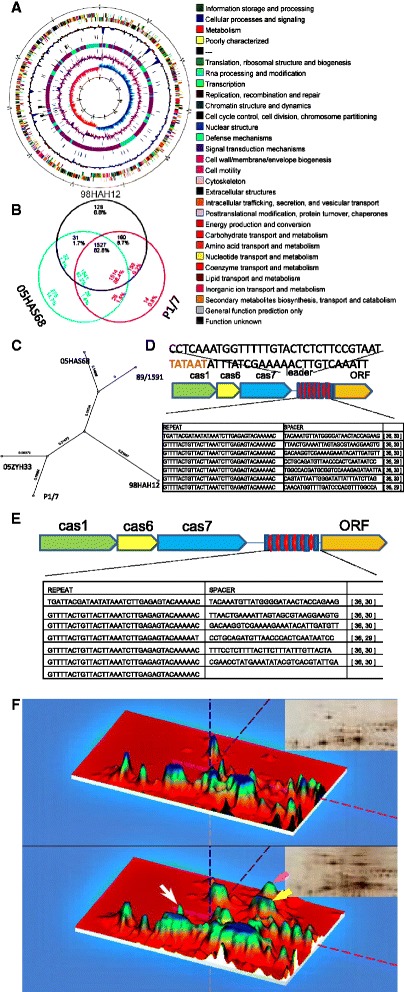
Circular representation of the 05HAS68 genome and *in silico* comparisons between *S. suis* genomes. **a** The outer circle shows the scale (bp). From in to out indicate that *circle 1*: GC-skew (*blue*: > 0; *light coral*: < 0), circle 2: G + C content (avg = 41%), *circle 3*: duplicated genes, *circle 4*: colinear blocks (*blue*: unique to strain68; *purple*: colinear with strain05), *circle 5*: unique genes (inner: on plus strand; outer: on minus strand), *circle 6*: gene density (avg = 85%, win_size = 5 kb; *blue*: below avg level; *red*: above avg level), *circle 7*: cds (color: see COG legend; inner: on plus strand; outer: on minus strand), *circle 8*: coordinates. **b** The 05HAS68, 98HAH33, and Sanger protein sets were compared using FASTA3. Numbers under the strain name indicate genes that are not shared with the other strains; values in parentheses are the number of proteins in each strain (excluding frame shifted and degenerated genes). Numbers in the intersections indicate genes shared by two or three strains. These are displayed in the color corresponding to the strains used as the query. Numbers in any given intersection are slightly different due to gene duplications in some strains. **c** Phylogenetic tree of *S.suis* serotype 2 strains based on genomic sequences. The CRISPR antiviral system of 05HAS68 (**d**) and 89/1591 (**e**). **f** Two-dimensional gel electrophoresis profile of silver stained whole-cell proteins from *S.suis* 2 strain 05HAS68 (*a*) and 05ZYH33 (*b*) grown to the mid-exponential phase. In the 3D image displayed, the important virulence proteins EF, MRP and SYL are indicated by color *arrow grey*, *black* and *white*

The strain 05HAS68 genome is a single, circular chromosome of 2,188,363 bp (Fig. 2a) with a GC content of 41.18%. Here, 1041 genes (82.2%) have homologs in all 3 genomes. Strain 05HAS68 shares 32 genes (1.7%) with 98HAH12 and 26 genes (1.4%) with P1/7. Finally, 275 genes (14.7%) are unique to 05HAS68 (Fig. 2b).

Except the 89 K large insertions in 05ZYH33 and 98HAH33, few differences were found in virulent strain P1/7 that has been reported by us. The 05HAS68 genome is similar in size, number of open reading frames (ORF), GC content, gene length and density to strains 05ZYH33 and 98HAH33, which were sequenced by our lab 2 years ago. However, the order of genes and genome fragments, gene components and a number of the CDS are quite different compared to the virulent strain (Table 1). Furthermore, the capsule plays an important role in virulence and dividing serotypes and 14 genes that control capsular biosynthesis were found to be identical between 05HAS68, 05ZYH33, 98HAH33 and P1/7.

**Table 1:** Genome features of 05HAS, 05ZYH33, 98HAH12 and P1/7

Feature	Genome			
	05HAS68	05ZYH33	98HAH12	P1/7
Length of sequence(bp)	2,188,363	2,096,309	2,095,698	2,007,491
G + C content	41.18%	41.11%	41.11%	41.30%
Repeat content length (%)	9032(0.41%)	9605(0.46%)	9357(0.45%)	0.43%
Simple repeats length(coverage)	411(0.02%)	351(0.02%)	243(0.01%)	284(0.01%)
Low complexity length(coverage)	1903(0.09%)	2339(0.11%)	2330(0.11%)	1927(0.10%)
Repeat element length	659	612	706	323(0.02%)
Small RNA #(length, coverage)	30(6, 059, 0.28%)	30(6, 303, 0.30%)	30(6, 078, 0.29%)	28(6053, 0.30%)
ORF (Length in Genome)	1,861,062	1,766,700	1,771,236	1,726,753
Feature				
ORF #	1874	1824	1836	1719
Genome coverage (%)	85.04%	84.28%	84.52%	86.02%
Genome density (genes/Kb)	0.86	0.87	0.88	0.86
Average gene length (bp)	993	969	965	1005
Max gene length (bp)	7272	6822	5910	6285
Function				
ORF with assigned functions	1741	1801	1809	1594
Conserved hypothetical protein	83	16	20	95
ORF without database match	50	7	7	30

Two-component gene regulatory systems (TCSs) have been well studied in many pathogenic bacteria and are known to regulate expression of many key virulence factors, such as extracellular toxins [19–23]. The two genomes of the *S.suis* 2 Chinese virulent strains, 05ZYH33 and 98HAH33, have 15 of the same conserved TCSs. Except that two of them are located in the specific 89 K GEI of the Chinese virulent strains [12], are the same as those in strain P1/7. However, only nine of 15 TCSs were found in the avirulent strain, 05HAS68 (Table 2). The results of sequence and proteomic analysis indicated that some major putative virulence factors, such as EF and SLY, are absent in strain 05HAS68 (Table 3, Fig. 2f). Analysis of the virulence factor MRP, in both strains 05HAS68 and 89/1591 using clustalw and antigenic peptide prediction, showed a large number of mutants on amino acid sequences and antigenic epitopes (Additional file 1: Figure S1).

**Table 2:** Analysis of virulence-associated genes from 05HAS68 and 05ZYH33 strains

Function category	Gene name	
	05HAS68	05ZYH33
Adherence/Colonization factors		
Fibrinogen binding protein (*fbp*)	PSN_CDS03155	SSU05_1492
	PSN_CDS01399	SSU05_1663
Muramidase-released protein (*mrp*)	PSN_CDS00463*	SSU05_0753
Agglutinin receptor	PSN_CDS02376	SSU05_0965
Adhesin	PSN_CDS04016	SSU05_2068
	PSN_CDS00076	SSU05_0112
	PSN_CDS00520	SSU05_0330
Autotransporter adhesion	PSN_CDS00785^*^	SSU05_0474
Cell surface factors		
Extracellular protein factor (*ef*)	-	SSU05_0177
	-	SSU05_0178
Exotoxins		
Hemolysin III homolog	PSN_CDS02424	SSU05_0994
Putative hemolysin	PSN_CDS01385	SSU05_1668
	PSN_CDS01413	SSU05_1653
Suilysin	-	SSU05_1403
Siderophores		
ABC-type Fe^3+^ siderphore transport system		
Permease component	PSN_CDS03427	SSU05_0646
	PSN_CDS03424	SSU05_0647
ATPase domain	PSN_CDS02486	SSU05_1031
ATPase component	PSN_CDS03419	SSU05_0650
Miscellaneous		
Tetracyline resistance protein (tetM)	PSN_CDS02364^*^	SSU05_0922

*The gene is largely mutated

**Table 3:** Two component signal transduction systems identified in the genome of different *S. suis* 2 strains

TCS	Predicted function	Demonstrated role in virulence of *S. suis*2	*S. suis*2 strain			
			05ZYH33	98HAH12	05HAS68	P1/7
LiaS/LiaR	Cell wall Stress response, antimicrobial resistance	No	1	1	1	1
NisK/NisR	Lantibiotic production	No	1	1	-	-
SalK/SalR	Lantibiotic production	Yes	1	1	-	-
CiaH/CiaR	Competence and antibiotic susceptibility	No	1	1	1	1
YycF/YycG	Essential for growth	No	1	1	1	1
YxdK/BecR	antimicrobial resistance	No	2	2	2	2
VanR/VanS	Vancomycin resistance	No	1	1	1	1
YocF/YocG	Thermosensing	No	1	1	1	1
CsrR	Virulence	Yes	1	1	1	1
YesM/YesN	Unknown	No	1	1	-	1
Orphan rr of the LytR family	Regulation of murein hydrolase activity	No	2	2	1	2
RevS	Virulence	Yes	1	1	-	1
VirS/VirR	Virulence	Yes	1	1	-	1

Since it is hard to find the real ancestor of *S.suis* 2, the last common ancestor (LCA) of the *S.suis* genome was reconstructed by identifying the homologous genes among the 05ZYH33, 98HAH33, 05HAS68, P1/7 and 89/1591 strains, and assuming that the genes of the LCA were still present in at least two strains and that the special genes in each strain were genes, which were gained. The genes were included in order to construct the LCA genome and to study evolutionary phenomenon, such as rearrangement, duplication, and gain or deletion in each ongoing genome based on the available information, though this arbitrary classification cannot produce the exact genome. In total, 1920 orthologous clusters (ORs) were obtained in these five strains, and cumulative study showed that about 37 orthologous genes may have been reduced with the addition of more strains (Fig. 2b, Additional file 1: Table S1, Table 2). The actual gene number may vary in different strains due to gene duplication. Compared to the LCA, the five strains were missing 192, 181, 223, 235, and 434 ORs, but had added 80, 336, 121, 9, and 155 genes. To evaluate the relationships among strains, a dendrogram was constructed based on noncore genic differences. As expected, strain 05HAS68 and strain 89/1591 from North America, was the most closely related pair (Fig. 2c).

The clustered regularly interspaced short palindromic repeat (CRISPR) presents an interesting genetic marker for comparative and evolutionary analysis of closely related bacterial strains [24, 25]. Analysis of the *S.suis* 2 05HAS68 complete genome sequence revealed a locus of 1767655–1768150 bp, having a typical CRISPR organization: eight repeats of 36 bp, separated by unique sequence spacers of 30 bp. A clustering of the cas1, cas6 and cas7 genes (stc: str0658, str0659 and str0660) was localized upstream of the locus. A leader sequence, which contained an AT-rich sequence, was located 5’to the CRISPR loci, directly adjoining the first repeat (Fig. 2d). A similar structure was found in contig 269 of 89/1519 (gi|80977822|gb|AAFA02000001.1|), though one repeat of 36 bp was absent (Fig. 2e). CRISPRs were not found with CRISPR recognition tool (CRT) when examining strains 05ZYH33, 98HAH33 and P1/7 [18].

### Genome feature with large scale arrangement

**Fig. 3:**
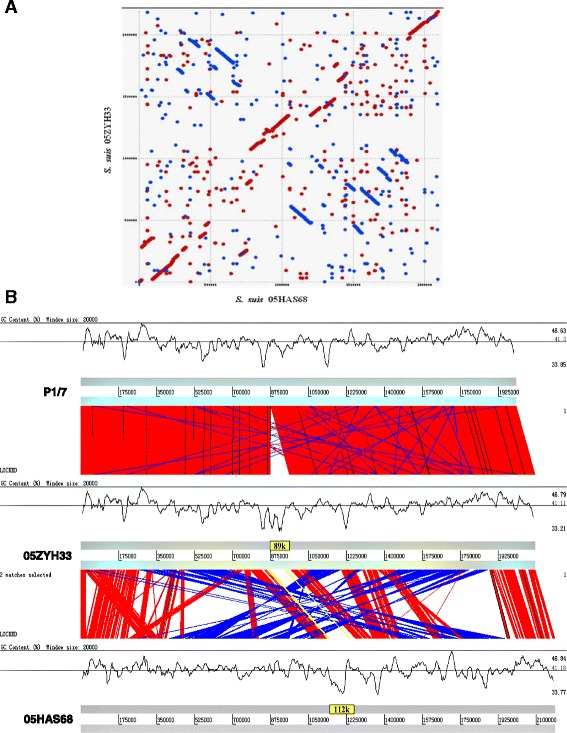
Whole-genome alignments between the P1/7 and 05ZYH33 strains with the 05HAS68 strain are shown by using MUMmer or ACT. **a** Dotmatch analysis in MUMmer. 05HAS68 or 05ZYH33 genome sequnce was plased X or Y axes. The red dot indicates that the match DNA sequence is in the same direction and the blue dot indicates the opposite direction. **b** The G + C content of genomes are shown by 20000-bp sliding window plot and the *thin grey line* tracing the average G + C content

Whole-genome alignments of 05HAS68 with 05ZYH33 or a Sanger P1/7 display included all commonly used rearrangement operations that affect a permutation, including reversals (also called inversions), transpositions, and block interchanges (i.e., generalized transpositions). Large chromosomal rearrangements, including both inversions and translocations and at least seven large inversions rearrangement locations were observed, when comparing 05HAS68 to the 05ZYH33 and P1/7 strains. These inversions seemed to create an “X-shaped” like alignment, which likely resulted from chromosomal inversions that pivoted around the origin and terminus many times (Fig. 3a). However, in the *S.suis* serotype 2 strains, the devil of account genes from phages, but no useful information was acquired to hint that contain prophage in these strain chromosome with prophage prediction software phage finder [26], except a few transposases local in these fragment upstream or downstream such as on sequence of 05HAS68 chromosome site at bases 630703 to 788878 (IS111A), bases 868905 to 920358 (IS204) or bases 1772301 to 1887337. Despite numerous rearrangements and IS elements, the 05HAS68 genome has remained remarkably stable during over a period of 2 years of cultivation in vitro (data not shown).

Most of the genes from prophages were found in these genomes differed in their DNA sequences, suggesting that they were acquired (and then often degraded) after separation of the bacterial lineages. However, at the DNA sequence level the genomes could still be aligned over essentially the entire chromosome (data not shown).

### Structural characterization of a GEI

**Fig. 4:**
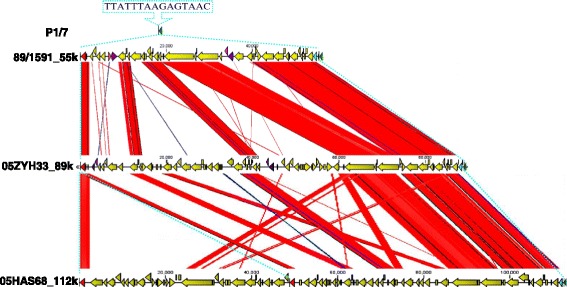
Analysis of site-specific genomic island. Colored column represents gene homologues. *Green* = RPL7/12, *black bar* = 15 bp repeat sequence, *red* = integrase, *blue* = replication initiator, *blue* = 89 k, *violet* = 2-component signal transduction systems, histidine kinase (*deep*) and DNA binding regulatory protein (*light*)

We previously reported that a candidate GEI of ~89 kb in length, which is related to virulence, was investigated at the genomic level [12]. This GEI was found to be site-specifically integrated in the 3’ end of the ORF L7/12 of *S.suis* 05ZYH33 and 98HAH33. This was compared with a strain very closely related to P1/7, which does not carry any element integrated in the 3’ end of L7/12. As one novel type GEIs, they contain 15 bp (TTATTTAAGAGTAAC) insertion sequence (IS), which was present in 5’ terminal of highly conserved gene L7/L12. This interesting mosaic structure suggests that this region of the genome is a hot spot for lateral GEI transfer. The franking 89 kb genomic island from this IS element in the chromosome of *S.suis* 2 strains 05ZYH33 and 98HAH33 was noticeable. The IS element also emerged in the 89/1591 strain genome. Since the genomic sequence of strain 89/1591 is unfinished, a novel genomic island, named 55 k, was predicted and assembled from four contigs (gb: AAFA02000004.1, gb: AAFA02000007.1, gb: AAFA02000008.1, gb: AAFA02000134.1). Comparing and mapping this IS element between strains 05ZYH33 and 05HAS68 showed that a large 112 k DNA fragment was inserted in the hot spot. In these regions, G + C content differ significantly from that of the overall G + C content of the host chromosomes (Fig. 3b). Besides franking the fragment, one of the IS elements was located in the 112 k fragment and divided it into 48 k and 64 k. This result suggests a potential tandem integration of GEIs. GEIs 89 k, 112 k and 64 k have identical features, which were found to be integrated at the 3’ end of the chromosome L7/L12 gene, presented integrase at 3’ terminal and replication initiator A at 5’ terminus of the GEI and mosaic structures of several individual acquisitions (Fig. 4). As previously reported, the 89 k GEI carries some genes or gene clusters expressing virulence related proteins and regulators [12] and the 48 k GEI carries a novel system responsible for L-fucose metabolism and the 64 k genetic loci encodes galactose metabolism. Both the 48 k and 64 k GEIs carry two components (*Vir*B4 and *Vir*D4, respectively) from the type IV secretory pathway. Analysis of the 48 k GEI construct showed that it was truncated to lose the replication initiator A gene at the end of the 5’ terminus.

### Evaluation of protective immunity of a piglet against *S. suis* strain 05ZYH33

**Fig. 5:**
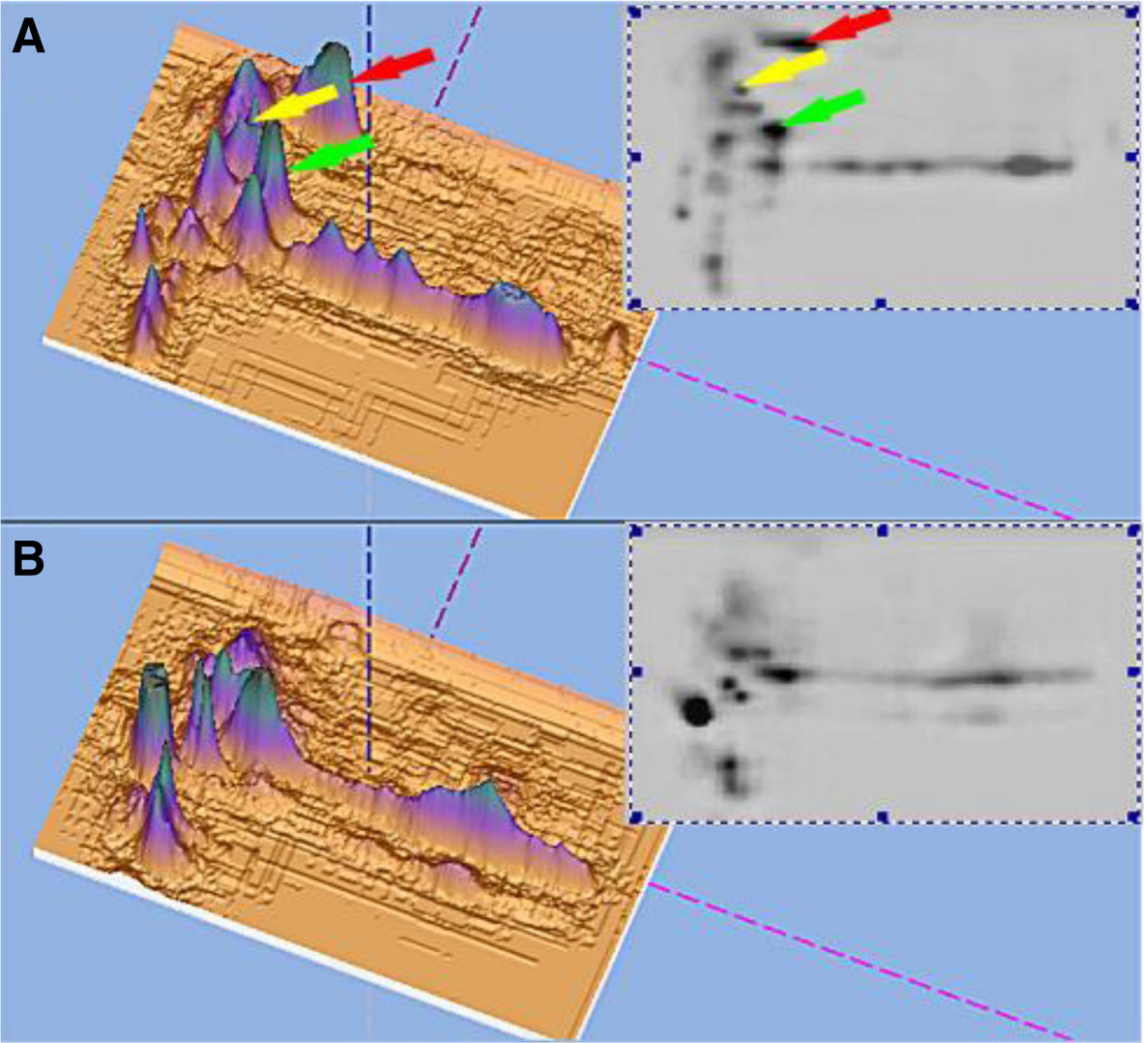
Comparison of whole-cell proteins extracted from *S.suis* 2 strain 05ZYH33 by 2-dimensional Western blotting using anti-sera pooled from pigs in the convalescent-phase, which had been clinically infected with the *S.suis* 2 strain 05ZYH33 (**a**) or vaccinated with 05HAS68 (**b**). The MRP, EF and SLY proteins are indicated by *red*, *yellow* and *green arrows*

Piglets were vaccinated with either the 05HAS68 or 05ZYH33 strain or were used as a control. After two vaccinations given intravenously, via the ear vein, the piglets were challenged with 1 × 10^8^ CFU of the 05ZYH33 virulent strain administered intraperitoneally (i.p.). All of the piglets (100%) immunized with the 05HAS68 avirulent live vaccine strain survived, whereas 100% of unvaccinated control piglets died after challenge with 05ZYH33. The time to death/survival ratio of piglets immunized with the strain 05HAS68 was significantly (*P* < 0.001) greater than that of nonimmunized piglets (Fig. 5a).

A 2-D Western blot approach was used to compare and visualize the reaction of the whole 05ZYH33 bacteria proteome to swine sera from convalescent-phase piglets with 05ZYH33 infection or piglets vaccinated with 05HAS68. After vaccination in the field trial, the same specific antibodies against the 05ZYH33 strain were induced and compared with the convalescent phase 0f 05ZYH33 infection except with anti-EF, MRP and SLY (Fig. 5). The Wilcoxon signed ranks test was used to analyze the level of serum specific IgG to *S.suis* in anterior-posterior vaccination 05HAS68. The result showed that the level of anti-*S.suis* specific IgG in serum from piglets vaccinated with 05HAS68 was significantly increased P (Z) < 0.05. The reactive IgG titers were more than 1: 5000.

**Fig. 6:**
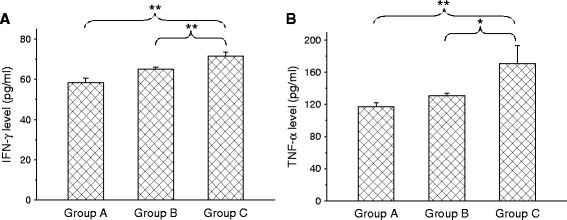
Level of cytokine IFN-γ and TNF-α from different phases of plasma: Group A indicates the cytokine value from naïve piglets. Group B and C indicates cytokine values from plasma after 48 h after vaccination with strain 05HAS68 or from piglets (*n* = 6) that had been vaccinated twice with strain 05HAS68 and then challenged by an intravenous injection of virulent strain 05ZYH33 2 days later. Values are arithmetic means ± standard errors of the means. *, *P* < 0.05; **, *P* < 0.01

The plasma levels of TNF-α and IFN-γ, TNF-α and IFN-γ were not significantly raised (*p* < 0.05) 2 days after vaccination with the 05HAS68 strain. Compared with naïve piglets, the levels of TNF-α or IFN-γ in the peripheral blood of 05HAS68 vaccinated piglets challenged with 05ZYH33 were significantly increased (*p* < 0.01) (Fig. 6).

### Specific T cells transfer protection

**Fig. 7:**
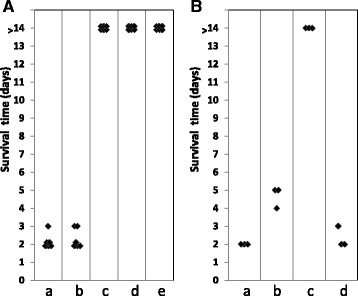
Observed survival time of piglets. **a** Protection induced by vaccination of piglets with the live avirulent *S.suis* serotype 2 strain 05HAS68. (*a*) and (*b*) The piglets were challenged intravenously with stain 05ZYH33 and 98HAH12 as positive control.; groups (*c*) and (*d*) on day 7 after vaccination with 10^9^ colony forming unit (CFU) of avirilent 05HAS68, the piglets were injected with the same bacterial concentrations of 05ZYH33 or 98HAH12, separately; (*e*) The piglets were injected only with stain 05HAS68 as negative control. **b** Observed protection by transfer of active specific T cell against *S.suis* to thepiglets (4 piglets/group). (*a*) The piglets were challenged intravenously with stain 05ZYH33 as positive control; (*b*) the piglets were transfused intravenously active specific T cell (1.8 × 10^9^ cells) from the piglets were vaccined with stain 05HAS68 or (*c*) active specific T cell (1.8 × 10^9^ cells) and plasma (5 ml) mixture from the piglets were vaccined with stain 05HAS68; (*d*) the piglets were transfused intravenously T cells (1.8 × 10^9^ cells) and plasma (5 ml) mixture from healthy naïve piglets as negative controls. Each data point represents one piglet

Active specific T cells were obtained from the peripheral blood of piglets, from fraternal multiple births that was vaccinated with the live 05HAS68 avirulent strain, and activated via heat inactivation in vitro. These T cells were then transferred to 05ZYH33-infected piglets. The results showed that these T cells could significantly delay the time of death (48–72 h) in the 05ZYH33-infected piglets. Interestingly, the results in piglets injected with the mixture of these T cells and plasma from an immunized animal were similar to those observed in vaccinated piglets, which survived without any obvious clinical symptoms until the end of the experiment (14 days after infection with the 05ZYH33 virulent strain, Fig. 7b).

## Discussion

Chinese isolates of highly pathogenic *S.suis* 2 caused two recent large-scale human epidemics and caused a large number of pig deaths in China. The mechanisms involved in the pathogenesis and virulence of *S.suis* 2 are not completely understood. Attempts to control the infection have been hampered by lack of an effective vaccine. The identification and characterization of a potential live avirulent vaccine is one of the strategies and prerequisites for development of an effective vaccine.

At least 10% of swine in Germany carry an *S. suis* strain [27], and it is likely that the percent is higher in China. As the most prevalent among the invasive porcine and human isolates, the complete genome sequences from a few virulent *S.suis* 2 strains have been reported, including P1/7, 89/1591, 05ZYH33 and 98HAH33; however, the characterized genomes of avirulent strains, which comprise the overwhelming majority of *S.suis* 2 strains, are almost completely unknown.

The current study reports on an avirulent *S.suis* 2 strain, 05HAS68, which has similar basic biological features as Chinese isolates of highly pathogenic *S.suis* 2, with the exception of hemolytic activity in vitro. Proteome detection indicated that the 05HAS68 strain lacks some major putative virulence factors, such as EF and SLY. MRP as an important virulent factor, compare the amino acid sequences of MRP from strain 05HAS68, P1/7, 89/1591, and 05ZYH33, the protein from strain 05HAS68 and 89/1591 emerge large mutant. Analysis and prediction suggest that this mutant causes a change of potential biological function and antigenic epitopes. Analysis of the 05HAS68 genome indicates that it lacks six of 15 TCSs compared with the 05ZYH33 strain. At least three of the missing six TCSs relate to the virulence of *S.suis* 2. Comparing the genic contents of the five strains suggests that the total number of pan-genes in the *S.suis* 2 species is around 3880 and the total number of core genes is around 1290. The pan-genome size of *S.suis* 2 has not been estimated very well since only five sequences are currently available. When the genomes were analyzed together, fewer than 70% of all core-genes were conserved among all strains. When the genomes of individual strains were evaluated, 15 to 35% of the core-genes were noncore. Strain 05HAS68 and the typical North American virulent strain, 89/1591, belong to one branch of the noncore genes tree and strains 05ZYH33 and P1/7 are on another branch. This suggests that strains 05HAS68 and 05ZYH33 are different than the most recent common ancestor.

Previous research presented a comparative genomics approach to assess levels of recombination and positive selection pressure in the core-genome and pan-genome size for each of the Streptococcus taxonomic groups. The results suggested that *Streptococcus* is the greatest positive selection pressure, has the largest number of loci uniquely selected, and the lineage that had the greatest amount of gene gain and loss. This positive selection pressure means that *S.suis* 2 is likely to shuttle back and forth between pig and human hosts. We certainly concur with these authors that much of gene gain and loss of *S.suis* 2 likely reflects species-specific adaptation [5].

Comparing present genomes to the ancestor genome reveals clues to their virulence and environmental adaptation via four major evolutionary paths including genome rearrangement, deletion, duplication and horizontal gene transfer. The flexibility of genomes and examples of DNA rearrangement and their roles in gene regulation have been documented in different prokaryotes [28–30]. The whole-genome alignments between P1/7, 05ZYH33 and 05HAS68 displayed numerous DNA fragment inversions and transpositions in their chromosomes. The DNA rearrangements altering gene expression has been reported [29]. These inversions and transpositions chequered with *S.suis* 2 strains whether change the expression of virulent or relate genes, which deserve our further study… Previous studies from our laboratory, using two genomic sequences (05ZYH33 and 98HAH33) from clinical strains, identified novel genes that are not present in the Sanger P1/7 strain and suggested a GEI insert in the chromosome. In the current study, the genomes of four *S.suis* 2 strains were analyzed, and a similar GEI structure was found in strains 05HAS68 and 89/1591. Numerous integrative elements (e.g., prophages and/or integrative conjugative elements) integrate into the 3’ end of genes encoding tRNAs, though their sequences remain unmodified by the integration. Other integrative elements (e.g., most of the conjugative transposons) integrate into several or numerous sites [31]. Only a few elements site-specifically integrate into the 3’ end of protein-encoding genes. The substitution sequence is then generally only similar to the original one [28]. Similar to one novel type GEI, these sequences insert into a chromosome by site-specific integration at the 3’ terminal dependent 15 bp direct repeat sequence of an L7/L12 gene and require a functional integrase gene. It is very interesting that these similar GEIs carrying gene clusters, which are very different in function. A few of the strain-specific genes are in the 89 k GEI, although they do not show the classical features of pathogenicity islands, are flanked by insertion elements and display an atypical nucleotide composition, suggesting possible acquisition through horizontal transfer for adoptive evolutionary response to new hosts. Our current knowledge of the impact of mobile genetic elements, as GEIs, in their hosts comes primarily from pathogenicity islands in which bacteriophages, plasmids and transposons act as carriers of genes encoding toxins, effector proteins, cell wall modification enzymes, fitness factors, and antibiotic and heavy metal resistance determinants in pathogenic bacteria. Much less is known about the diversity and role of GEIs in nonpathogens, in which these elements may enable their hosts to adapt to changing environmental conditions or colonize new ecological niches [32]. As avirulent bacterium colonize in the nasopharynges of healthy pigs only, the 05HAS68 genomic 48 k GEI carries a novel L-fuculose metabolic system in *S.suis* 2 to adapt to this environmental parameter and the 64 k GEI carries a homeo-gene cluster, which is a classical lactose metabolism system. The 48 and 64 k GEI of strain 05HAS68 couple and assemble a larger 112 GEI. As the 48 k construct emerged it was truncated and lost the replication initiator A gene at the end of the 5’ terminus. It has been suggested that the 112 k construct evolved by site-specific accretion from a composite structure of GEIs. Although the 112 k GEI from avirulent strain 05HAS68 exhibits a partial similarity to 89 and 55 k GEIs from virulent strains, the virulent strains also carry large niche-specific genes that could putatively affect the survival of strain 05HAS68 in natural habitats. In addition, when strain 05ZYH33 were repeatedly inoculated and cultured for a long time in THB medium, the 89 k GEI was lost from the chromosome and the circular 89 k DNA was detected in the cells [33]. This supports a general hypothesis that GEI contributes to host fitness. Under the same culture conditions, a loss of 112, 48, or 64 k GEI has not been observed in the 05HAS68 strain. Possible reasons are either that the cells cultured in THB had some similarities with their primary habitat or that fitness was not lost due to interference by the CRISPR defense system; however, the reason(s) are not fully understood.

Indeed, to date, no successful vaccine against *S.suis* has been produced and heat-killed bacterial preparations have failed to induce protection. Because *S.suis* 2 commands such a large genic diversity, it is almost impossible to depend on 1 or even several virulence factors from one *S.suis* 2 strain to induce complete immunological protection for each of the *S.suis* 2 pathogenic strains. The heat-killed bacteria can be quickly cleaned by phagocytes and are not enough to induce specific immunological protection. In the current study, the live cells of the avirulent strain, 05HAS68, could be used for the immunization of swine, the natural host. It was completely cleared in the peripheral blood of the host until about 30 h, and thus had enough time to induce specific immunological protection. Recent studies suggest that the leading vaccine candidate, which primarily stimulates antibody-mediated humoral immunity, may not suffice. T cell-dependent cellular immunity comprises a second means by which vaccines prime long-lived protection against virulent bacterial pathogens [34].

Our results showed that the high titer IgG serum from piglets immunized against the avirulent strain could recognize most proteins of the tested virulent strain. Analysis of cytokines in the serum after 05HAS68 vaccination and 05ZYH33 challenge indicated that the levels of TNF-α and IFN-γ were significant increased. It has been reported that CD4 responses are associated with strong cell-mediated inflammatory responses, which may be favorable for pathogen elimination from the host [35]. The CD4 T cell is a major cellular source of TNF-α and IFN-γ. Mice lacking IFN-γ have been shown to be more susceptible to pneumococcal infection and administration of IFN-γ can enhance survival of mice after pneumococcal challenge [36, 37]. However, TNF-α is a double-edged sword; it is a proinflammatory cytokine that can promote protective immune responses against a variety of pathogens, but may also cause inflammatory host injury under certain conditions [38].

This study found that co-transference of active T cells and plasma from pigs of vaccinated with strain 05HAS68 provided global protection to pigs challenged with the 05ZYH33 virulent strain; however transferring one of the two were insufficient for providing immunological protection. This result suggests that plasma contains specific antibodies and that the bactericidal activity of T cells against *S. suis* is very important, because T cell immunity may also provide antibody-independent protection against *S.suis* 2 infection. Results of this study suggest that a naïve avirulent live cellular vaccine that promotes both humoral and cellular immunity will most effectively combat the highly virulent 05ZYH33 strain and has important implications for the development of an effective vaccine to protect pigs against *S. suis* infection.

## Conclusion

In conclusion, comparison of *S.suis* 2 genomes showed massive scale arrangement, which can potentially alter genes expression, an enormous amount of single gene gain and loss, and GEIs that carry different functional gene cluster inserts for *S.suis* 2 species. As *S.suis* 2 has the largest genic diversity, it is almost impossible to depend on using one or even several virulence related substances as vaccines to induce global immune protection for all *S.suis* 2 pathogenic strains. In view of these results, that specific protection was induced from humoral and cellular immunity via vaccination with the avirulent strain, 05HAS68, it is suggested that research strategies for vaccine design and development be reconsidered using model strains for testing.

## Additional file

Additional file 1: Figure S1. Comparing of the MRPs from four strains of *S.suis* 2. (A) Alignment of the amino acids sequences of MRP. (B) Phylogenetic comparison of MRP proteins. Figure S2 A novel system responsible for L-fucose metabolism from the GEI of 48 k. (A) L-fucose metabolic gene cluster. (B) Illustration of L-fucose metabolic pathway. Table S1 Prediction of antigenic peptides of MRPs from four strains of S.suis 2. (DOCX 552 kb)

## Abbreviations

ACT: Artemis Comparison Tool
BSA: Bovine serum albumin
CDS: Coding sequences
CRISPR: Clustered regularly interspaced short palindromic repeats
EF: Extracellular protein factor
ELISA: Enzyme linked immunosorbent assay
FASTA: It is a DNA and protein sequence alignment software package first described
GEI: Genomic island
IFN: Interferon
LCA: Last common ancestor
MRP: Muramidase-released protein
OD values: Optical density values
Ors: Orthologous clusters
PBS: Phosphate-buffered saline
PVDF: Polyvinylidene fluoride, or polyvinylidene difluoride
Sly: Suilysin
SPA-HRP: Staphylococcal protein A labeled with horseradish peroxidase
STSS: Streptococcal toxic shock syndrome
TCSs: Two-component gene regulatory systems
TNF: Tumor necrosis factor
