# Erratum to: Genome-wide analysis of an avirulent strain that induces protective immunity against challenge with virulent Streptococcus Suis serotype 2

**DOI:** 10.1186/s12866-017-1061-z

**Published:** 2017-07-04

**Authors:** Jing Wang, Youjun Feng, Changjun Wang, Feng Zheng, Bachar Hassan, Liming Zhi, Wenjuan Li, Yi Yao, Elaine He, Shibo Jiang, Jiaqi Tang

**Affiliations:** 1 Translational Medicine Center, PLA Hospital No. 454, Nanjing, 210002 China; 2 Department of Medical Microbiology and Parasitology, Zhejiang University School of Medicine, Zhejiang, Hangzhou 310058 China; 3 Department of Epidemiology, Medicinal Research Institute, Nanjing Military Command, Nanjing, 210002 China; 4 University of North Carolina, Chapel Hill, NC USA; 5 The Warren Alpert Medical School of Brown University, RI02912, Providence, USA; 6 PLA Research Institute of Clinical Laboratory Medicine, Nanjing General Hospital, Nanjing Military Command, Nanjing, 210002 China; 7 Lindsley F. Kimball Research Institute, New York Blood Center, New York, NY 10065 USA

## Erratum

After the publication of the original article [1], discrepancies were discovered in the title of the manuscript. The title of this manuscript should read *“Genome-wide analysis of an avirulent strain that induces protective immunity against challenge with virulent Streptococcus suis serotype 2”*. This has now been acknowledged and corrected in this erratum.
