# Supplementary material for: Genome-wide analysis of a avirulent and reveal the strain induces pro-tective immunity against challenge with virulent Streptococcus suis Serotype 2
Source: BMC Microbiol. 2017 Mar 14;17:67. doi: 10.1186/s12866-017-0971-0 (PMC5351164; doi:10.1186/s12866-017-0971-0)
Supplement: Additional file 1: Figure S1. — Comparing of the MRPs from four strains of S.suis 2. (A) Alignment of the amino acids sequences of MRP. (B) Phylogenetic comparison of MRP proteins. Figure S2 A novel system responsible for L-fucose metabolism from the GEI of 48 k. (A) L-fucose metabolic gene cluster. (B) Illustration of L-fucose metabolic pathway. Table S1 Prediction of antigenic peptides of MRPs from four strains of S.suis 2. (DOCX 552 kb) [file 12866_2017_971_MOESM1_ESM.docx]

**Supporting Information**

Figure S1 Comparing of the MRPs from 4 strains of *S.suis* 2. (A) Alignment of the amino acids sequences of MRP. (B) Phylogenetic comparison of MRP proteins.

Figure S2 A novel system responsible for L-fucose metabolism from the GEI of 48k. (A) L-fucose metabolic gene cluster. (B) Illustration of L-fucose metabolic pathway.

Table S1 Prediction of antigenic peptides of MRPs from 4 strains of *S.suis* 2

Figure S1

A

B


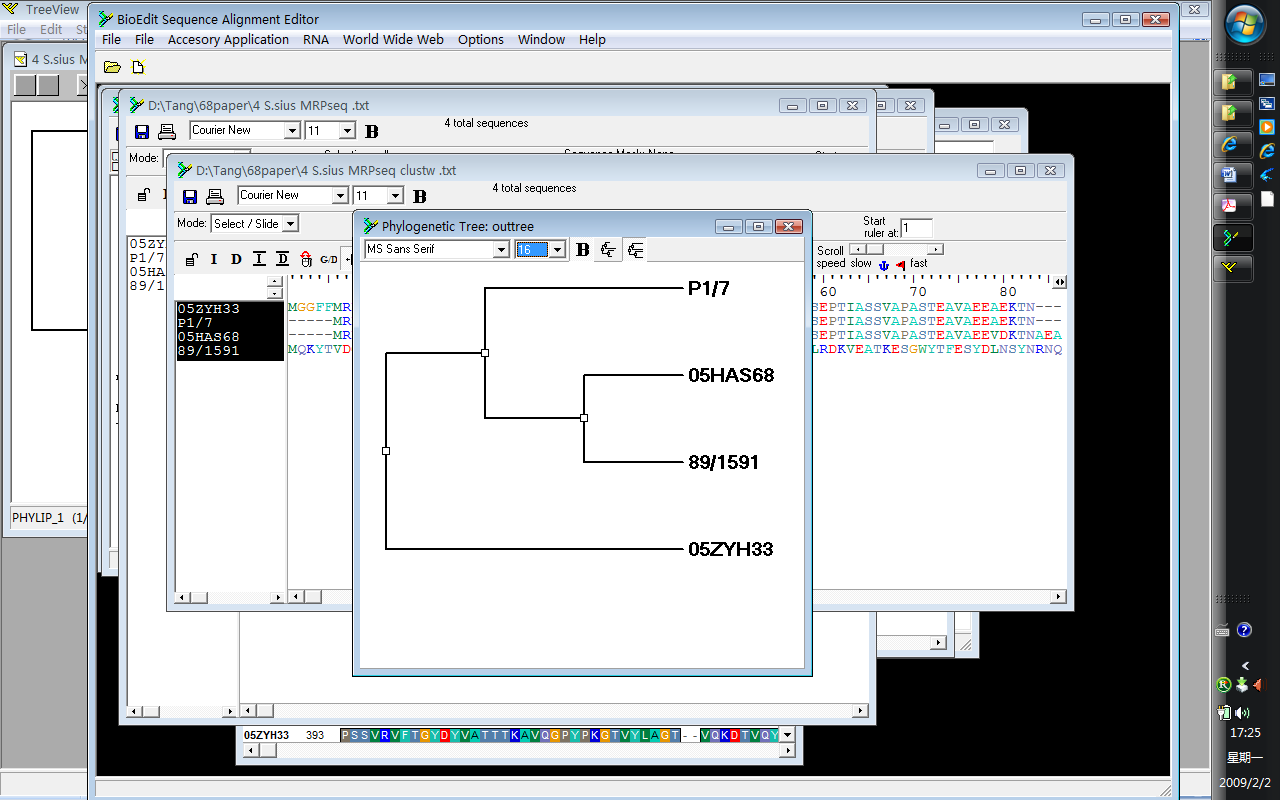


Figure S2

A


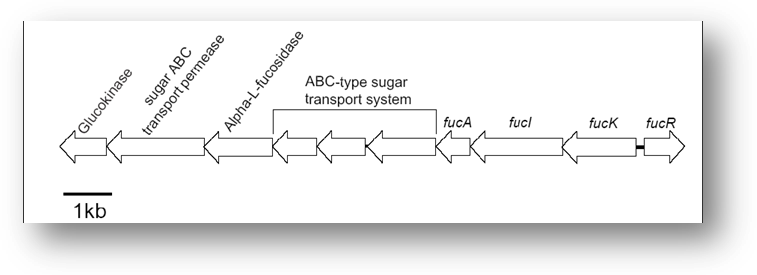


B


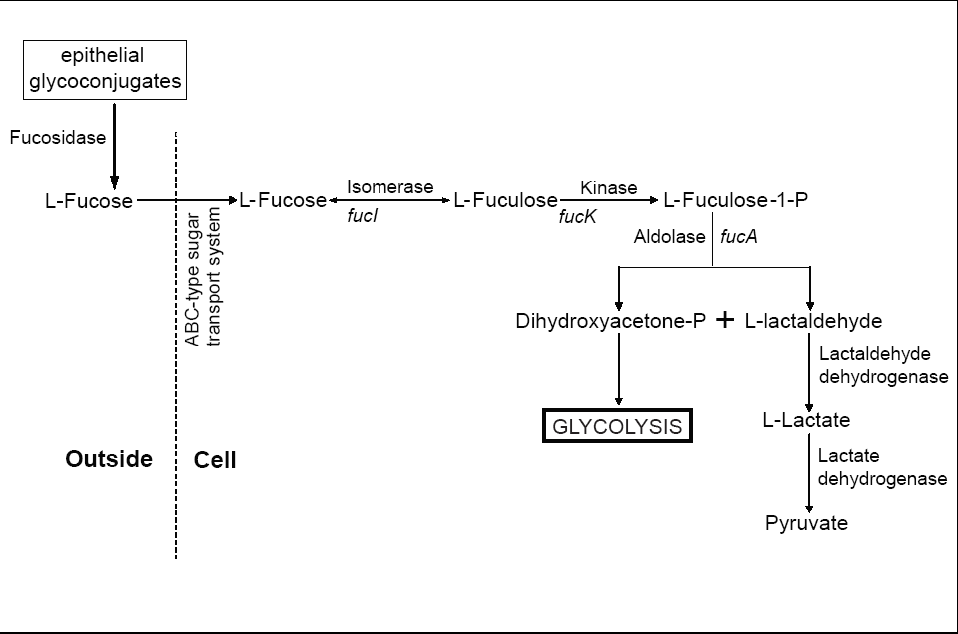


Table S1 Prediction of antigenic peptides of MRPs from 4 strains of *S.suis* 2
